# PROTOCOL: Voluntary work for the physical and mental health of older volunteers

**DOI:** 10.1002/CL2.190

**Published:** 2018-11-27

**Authors:** Trine Filges, Anu Siren, Torben Fridberg, Bjørn Christian, Viinholt Nielsen

## Background

### The problem, condition or issue

A fundamental public policy challenge in the Organization for Economic Co‐operation and Development (OECD) member countries^1^ has long been the issue of the increasing imbalance between the growing cohorts of older adults not working and the shrinking cohorts of adults in the age range of labour force participation (age range 20‐64)(OECD, 2015). The baby boom generation (born 1946 to 1955) grows older and social scientists and policy makers have taken an intense interest in how their aging and eventual retirement from the full‐time labour force will affect society. In only 15 years, the share of the population aged 65 and over in the OECD countries has increased by more than 3 percentage points; from 13 per cent in 2000 to more than 16 per cent in 2015 (OECD. Stat, data extracted August 10, 2016). The effect of an aging population on a country's societal support burden is often measured by the older dependency ratio, which is the ratio of the older population to the working‐age population. The OECD average older dependency ratio (ratio of individuals aged 65 and above to those aged 20‐64) has increased considerably over the last half century, from 17.9 in 1970 to 27.5 in 2015 (OECD. Stat, data extracted August 10, 2016). The problem is more pronounced in Europe than in the US; the older dependency ratio was 25.0 in the US in 2015 and as high as 31.3 in Europe^2^ in 2015 (OECD. Stat, data extracted August 22, 2016).

In addition to the effect of the large baby boom generation growing older, the average duration of expected years in retirement has increased. In 1970, men in the OECD countries spent on average 11 years in retirement and by 2014, this average had increased to almost 18 years (OECD, 2014). The increase for women has been from 15 years in 1970 to 22.3 years in 2014.

The increase in average duration of years in retirement is partly due to increased longevity and partly due to earlier retirement. Although the effective age of retirement (the average effective age at which workers withdraw from the labour force) decreased between 1970 and 2001, it slowly started to increase in 2004 (OECD, 2014). In 2014 the effective retirement age was on average 64.6 for men (63.2 for women) in the OECD countries; a bit higher in the US (65.9 for men and 63.2 for women) than in Europe^3^ (62.9 for men and 61.7 for women) (OECD. Stat, data extracted August 22, 2016). Life expectancy at the effective retirement age has also increased substantially during this period. Recently, the increase in longevity has been fairly equal to that of the effective exit age from the labour market, and potential years in retirement have stabilised (OECD, 2014).

By 2050, the population aged 65 and over in the US is expected to grow to almost 21 per cent and the older dependency ratio is estimated to increase to 38 (OECD. Stat, data extracted August 22). In Europe, the percentage of the population aged 65 and over is expected to grow to almost 29 per cent by 2050, and the older dependency ratio is estimated to increase to 55 (OECD. Stat, data extracted August 22). At a societal level, this growing imbalance raises serious concerns about the viability and funding of social security, pensions, and health programmes.

At an individual level, the concern is probably more that of aging well with the prospect of many years in retirement. The concept of aging well clearly implies maintaining health and effective functioning. Research suggests, however, that retiring for some carries the risk of a fast decline in health (Dave, Rashad & Spasojevic, 2008; Szinovacz & Davey, 2004). The reason may be that retiring deprives people of the deep‐seated needs they have for time structure, social contact, collective effort or purpose, social identity or status, and regular activity, which paid work generally provides (Jahoda, 1981; Jahoda, 1982; Jahoda, 1984). The absence of these latent supportive features of employment may be detrimental to the health of retired workers. In fact, according to Dave, Rashad and Spasojevic (2008), complete retirement leads to rapid declines in mental health, increases in illness, and increases in difficulty performing daily activities.

Complete retirement in an early age may thus threaten the ability of individuals to age well and societies as a whole aging well because of the societal burden resulting from health and functional limitations and associated costs. Several studies have demonstrated that subjective usefulness is strongly related to both physical and psychological health (Ranzijn, Keeves, Luszcz, & Feather, 1998; Ryan & Frederick, 1997; Ryff, 1989). The performance of other meaningful (for the individual) activities than working for pay may thus help maintain health and functional ability for older people.

Using US data from 1995 and 2005, Einolf (2009) predicted that the baby boom generation's rate of volunteering at the age of retirement (in 2015) would be higher than earlier generations' rate of volunteering. Combined with the large size of the baby boom cohort, Einolf concluded that the total number of older volunteers would increase. The prediction seems to hold true, at least for those countries where it has been possible to locate relevant numbers for the baby boom generation's rate of volunteering. In Canada, the rate of volunteering for those aged 65 and over increased from 32 per cent in 2004 to 36 per cent in 2010 (Vézina & Crompton, 2012), and in Denmark the rate increased from 23 per cent in 2004 to 34 per cent in 2012 (Fridberg & Henriksen, 2014).

In the US, programmes have been initiated to integrate the aging population into voluntary work. Some programmes are organised in local non‐profit organizations, referred to as Senior Corps Programs. “Senior Corps” is a network of national service programmes that provides the opportunity for people aged 55 years or above to apply their life experience to meeting community needs (see www.seniorcorps.org/rsvp/senior‐corps‐programs‐2/).

Specific programmes utilized by Senior Corps include the Foster Grandparents Program, the Retired Senior and Volunteer Program (RSVP), and the Senior Companion Program. The idea of engaging older people is not entirely new, however; the Foster Grandparents Program began as a pilot programme in 1965, the Senior Companion Program began in 1968, and RSVP was created as a nationwide program in 1969. A more recent initiative is the Experience Corps, which began in early 1996. One of the mission statements of this programme is to “provide significant benefits for the older Americans who participate” (Grimm, Spring & Dietz, 2007, p. 26).

### The intervention

Volunteering is a complex phenomenon and spans a wide variety of types of activities, organizations and sectors. The intervention of interest in this review is formal volunteering. Formal volunteering can be described as voluntary, on‐going, planned, helping behaviour that intend to increase the well‐being of strangers, offers no monetary compensation, and typically occurs within an organizational context (Clary et al., 1998; Penner, 2002). We will define formal volunteering centred on four axes (as defined in Hustinx, Cnaan & Handy, 2010). These are:

1) Free will: Volunteering is a free choice; it is a voluntary action as opposed to a compulsory action.
2) Remuneration: The voluntary work offers no monetary compensation. There may be reimbursement for expenses incurred but otherwise the work is unpaid.
3) Intended beneficiaries: Volunteer work can be described as “unpaid work provided to parties to whom the worker owes no contractual, familial or friendship obligations” (Tilly & Tilly, 1994, p. 291). Thus, formal volunteer work typically benefits strangers and is often referred to as non‐obligatory helping (Omoto & Snyder, 1995).
4) Structure: Volunteering as defined here should involve planned and ongoing activities (as opposed to a spontaneous one‐time activity). Such planned and ongoing activities often occur in some type of organisational context (Penner, 2002). An organisation defines the content of the volunteer work and formulates some expectations to the volunteer, including the tasks of the volunteer worker. The organisation produces plans, recruits the volunteers, educates them if necessary, and leads them. Thus, the relations that occur in the voluntary work are formal and different from the informal relations that are found between friends and family members to whom the volunteer may feel obliged (La Cour, 2014).

An example of interventions eligible for inclusion is Senior Corps Programs, Foster Grandparents Program, the Retired Senior and Volunteer Program (RSVP), and the Senior Companion Program.

### How the intervention might work

Volunteering can play a significant role in people's lives as they move from work to retirement. According to Smith and Gay (2005), retirement is the trigger for volunteering for some older people, as it offers a ‘structured’ means of making a meaningful contribution in society once the opportunity to do so through work has been cut off. Some older people consider voluntary work as a way to replicate aspects of paid work lost upon retirement, such as organisational structure and time discipline (Smith & Gay, 2005). The same line of arguments for volunteering can be found in several other studies (see Chappell & Prince, 1997; Fischer, Mueller & Cooper, 1991; Greenfield & Marks, 2004; Newman, Vasudev and Onawola, 1985; Widjaja, 2010. Volunteering thus seems to provide a way of compensating for the losses due to retirement as identified by Jahoda (1981, 1982 and 1984), such as the need for time structure, social contact, collective effort or purpose, social identity or status, and regular activity. Several studies indeed argue that there is a potential health benefit to older volunteers and in particular retirees (Moen and Fields, 2002; Musick and Wilson, 2003; Young and Glasgow, 1998).

**Figure 1:**
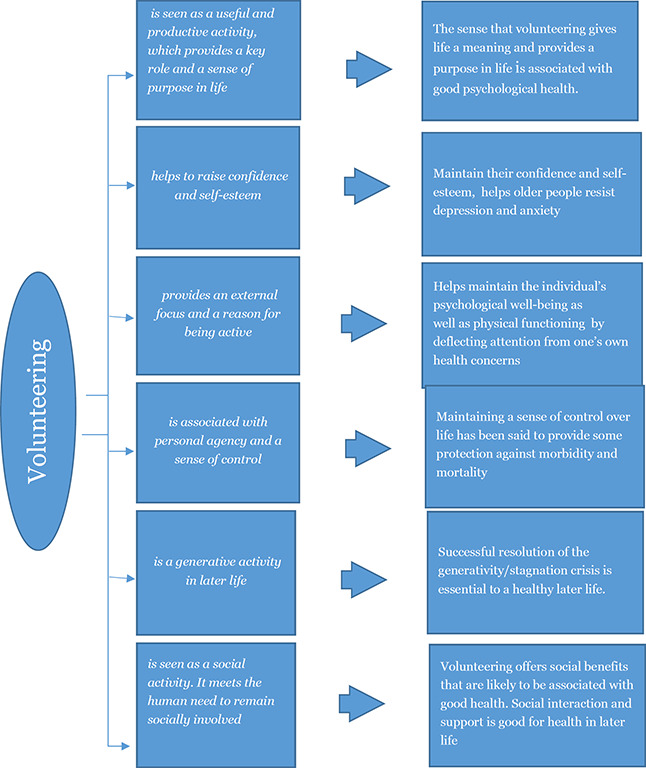
Mechanisms by which volunteering may affect health

The exact mechanisms and processes linking volunteering and health for older people has however not been sufficiently explored and may be very complex (Warburton, 2006). Using an in‐depth qualitative approach Warburton (2006) aims to explore this relationship. Warburton (2006) analysed the data, focusing on health. The respondents were not asked directly about the health impact of volunteering though. The study identifies six potential themes and the impacts on health is discussed. The six themes and their impacts on health is illustrated in Figure 1.

### Why it is important to do the review

Volunteering of the older adults seems to be on the increase and programmes designed specifically for this subpopulation are emerging. Volunteering may contribute to both individuals aging well and society aging well, as volunteering by the older adults at the same time relieves the societal burden if it helps maintain health and functionality for those who volunteer. It thus remains to be established to what extent volunteering impacts on the physical and mental health of those who volunteer.

Health status is often found to be an important predictor of volunteering among those aged 65 years or more, see for example Brown (2000) and Young and Glasgow (1998). The question that is important to answer is: Does good health predict volunteering or does volunteering improve health (or maybe both)? Studies that simply assess the association between voluntary work and health outcomes cannot answer this question. Research using appropriate controls and outcome measures can, however, provide some relevant evidence on whether engaging in voluntary work might cause good health outcomes on older people. It is vital that an appropriate comparison group is used to establish the direction of cause. Does volunteering make people healthier, or are healthier people more likely to volunteer? Likewise, it is vital that the health measures are objective. As stated in Wilson and Musick (1999, p. 153): “[C]ross‐sectional designs that use participants to self‐assess the impact of a volunteer program function as little more than market research for the agency concerned. Without a pre/post‐test design and a control group, and without more objective and generalizable outcome measures, little can be learned of the benefits of volunteering from these studies”. The same worries concerning reliance on cross‐sectional designs and self‐assessment of health to establish causality can be found in Lum and Lightfood (2005). Hence, considering the fact that the population under investigation in this review by nature volunteer into the intervention, we believe it is vital that an appropriate comparison group and access to relevant pre health measures and objective health measures are used to establish causality.

We are very clear that firm causal conclusions probably cannot be drawn from the studies we expect to include in the review, as we do not expect to find any studies based on randomised trials. However, a distinction can be drawn between studies that simply assess the association between voluntary work and health outcomes, and studies that control for important confounding factors, in particular pre health measures, and use objective health measures. Studies that control for important confounding factors and use objective health measures provide some evidence for considering possible causal effects. While conclusions about causal effects must be very tentative, it is important to extract and summarize the best evidence available.

An obvious question arises: is there any value in conducting a systematic review when it is likely that there are no trial based studies available? We think it is worthwhile as a systematic review may uncover high quality studies that may not be found using less thorough searching methods. Furthermore, if a systematic review demonstrates that high quality studies are lacking, this could encourage a new generation of primary research. Therefore, even though we expect not to find any trial based studies and only a few studies of voluntary work based on appropriate outcome measures and control group comparison, we still believe there is value in conducting the proposed review.

## Objectives

The main objective of this review is to answer the following research question: What are the effects of volunteering on the physical and mental health of people aged 65 years or older?

## Methodology

### Criteria for including and excluding studies

#### Types of study designs

It is hard to imagine that a researcher would randomise the allocation of people to volunteer work. We therefore anticipate that relatively few randomised controlled trials on the effects of volunteer work on the health of the volunteers will be found. However, in the unlikely event that a randomised controlled trial is found, it will of course be included in the review. In order to summarise what is known about the possible causal effects of volunteering, we will include all study designs that use a well‐defined control group. Non‐randomised studies, where voluntary work has occurred in the course of usual decisions outside the researcher's control, must demonstrate pre‐treatment group equivalence via matching, statistical controls, or evidence of equivalence on key risk variables and participant characteristics. These factors are outlined in the section *Assessment of risk of bias in included studies*, and the methodological appropriateness of the included studies will be assessed according to the risk of bias model.

The study designs we will include in the review are:

A. Randomised controlled trials (where all parts of the study are prospective, such as identification of participants, assessment of baseline, and allocation to intervention, and which may be randomised, quasi randomised or non‐randomised), assessment of outcomes and generation of hypotheses (Higgins & Green, 2008).
B. Non‐randomised studies (voluntary work has occurred in the course of usual decisions, the allocation to working voluntary and not working voluntary is not controlled by the researcher, and there is a comparison of ***two or more groups*** of participants).

#### Types of participants

The “intervention population” are people aged 65 or over who are engaged in formal voluntary work. Studies where the majority of participants are aged 65 or over, or where results are shown for subgroups of participants aged 65 or over, will be included. We will include voluntary workers of both genders and all nationalities who perform all types of formal voluntary work as defined in the Intervention section.

#### Types of interventions

The intervention of interest in this review is formal volunteering. Formal volunteering can be described as voluntary, on‐going, planned, helping behaviour that intend to increase the well‐being of strangers, offers no monetary compensation, and typically occurs within an organizational context (see the Background section). Informal ways of helping friends, neighbours, or relatives, such as running errands, providing transportation etc., which are typically motivated by an obligation to help intimate others, will be excluded.

The comparison population are people who are not engaged in formal voluntary work.

#### Types of outcome measures

The primary focus is on measures of health.

#### Primary outcomes

As primary outcomes we will include physical health outcomes as well as mental health outcomes. All measures of physical health outcomes reported in studies using a comparable control group have to be objective in order to be included as primary outcomes. As mentioned above Wilson and Musick (1999) highlight the problem with studies relying on self‐assessment of the impact of volunteering. Self‐assessment of health should not be confused with self‐reported measures. By self‐assessment we understand questions of the form: “Would you say the state of your health is excellent, good, fair, poor or very poor?” which will not be included as a primary outcome. On the other hand, we do not expect that measures of mental health outcomes be obtained via structured clinical interviews. Instead, we expect that self‐reported questionnaires be used to screen for probable mental disorders.

The use of different instruments of detection may be an important source of variation for the incidence of measured mental health outcomes. Measures of health have to be standardized to be included, see below. General scales of well‐being will be included if they are measured by standardized psychological symptom measures.

Examples of physical health primary outcomes include mortality, time until the onset of a serious disease (as for example a heart attack, stroke, cancer, arthritis), functional disability (measured by a standardized physical ability measure such as a difficulties in activities of daily living score (ADLs, see Katz et al., 1963)), or a difficulties in instrumental activities of daily living score (IADLs, see Lawton and Brody, 1969).

Examples of mental health primary outcomes include depression, anxiety and mental health‐related disability measured by standardized psychological symptom measures such as the Center for Epidemiological Studies Depression Scale (CES‐D), the Hopkins Symptom Checklist and the Medical Outcomes Study – Short Form.

#### Secondary outcomes

Although some researchers express concerns about using self‐assessed health measures (Wilson & Musick, 1999 and Lum & Lightfood, 2005), others argue that self‐assessed health can be a good predictor of mortality (Jylhä, 2009 and Jylhä, Volpato & Guralnik, 2006). If studies report self‐assessment of health, using questions of the form: “Would you say the state of your health is excellent, good, fair, poor or very poor?” they will be included as secondary outcomes.

#### Duration of follow‐up

Time points for measures considered will be:

- While actively engaged in voluntary work
- At cessation of volunteering to one year after cessation of volunteering
- More than one year after cessation of volunteering

#### Types of settings

The volunteer work may be done in all organisational contexts such as religious organisations, educational organisations, health organisations political groups, sports clubs, cultural organisations, senior citizen groups or related organisations.

Activities performed by individuals who, of their own accord, engage in the sustained, non‐obligated helping of strangers will, however, also be included. We are aware that it may be difficult to distinguish such activities from informal ‘helping out’. New forms of organising volunteer work are coming. An example of such an activity to be included (i.e., that is more than the informal helping out between friends and family members) is the on‐going volunteer work done under the auspices of ‘Venligboerne’ in Denmark. Venligboerne is an initiative that is managed by the civil society and people are linked together by a common identity and a common goal of creating an inclusive community for refugees. People can arrange, organise and volunteer in local^4^ joint initiatives such as establishing a café at the asylum centre to arrange large celebrations of festive seasons without being framed by an organisation with given structure (for more information see Kelstrup, 2016). If relevant studies of this kind of voluntary activities are identified, they will be analysed separately.

### Search strategy

Relevant studies will be identified through searches in electronic databases, governmental and grey literature repositories, hand search in specific targeted journals, citation tracking, contact to international experts and internet search engines. Following international databases will be searched:

- SocIndex
- PsycInfo
- EconLit
- Academic Search
- Science Citation Index
- Social Science Citation Index
- PubMed
- Medline
- Cochrane Library
- Social Care Online

### Grey literature search

Following grey literature resources will be searched:

UK Institute for Volunteering Research ‐ https://www.ncvo.org.uk/institute‐for‐volunteering‐research

Danish Institute for Voluntary Effort ‐ https://frivillighed.dk/

Danish National Research Database ***‐*** http://www.forskningsdatabasen.dk/en

Volunteer Bénévoles/Canada ‐ https://volunteer.ca/research‐resources

Corporation for National and Community Service ‐ https://www.nationalservice.gov/

Royal Voluntary Service ‐ https://www.royalvoluntaryservice.org.uk/

NBER Working Papers ‐ http://www.nber.org/papers.html

Open Grey ‐ http://opengrey.eu/

Google Scholar (specific for grey literature) ‐ https://scholar.google.com

Google searches ‐ https://scholar.google.com/

Further sources of grey literature might be added throughout the search process.

### Hand search

Five specific journals will be hand‐searched:

The Journals of Gerontology

American Journal of Public Health

Gerontologist

International Journal of Geriatric Psychiatry

Journal of Applied Gerontology

### Citation tracking

In order to identify both published studies and grey literature we will utilize citation‐tracking/snowballing strategies. Our primary strategy will be to citation‐track related systematic‐reviews and meta‐analyses. The review team will also check reference lists of included primary studies for new leads.

### Contact with international experts

We will contact international experts to identify unpublished and ongoing studies.

### Search terms

Down below is an example of a search string used to search PsycINFO. The search string will be modified accordingly to fit each database listed.

**Table:** 

**Search**	**Search Terms**
S15	S3 AND S11 AND S14
S14	S12 OR S13
S13	TI (old* OR aged* OR aging* OR elder* OR senior* OR life course* OR gerontolo*) OR SU (old* OR aged* OR aging* OR elder* OR senior* OR life course* OR gerontolo*) OR AB (old* OR aged* OR aging* OR elder* OR senior* OR life course* OR gerontolo*)
S12	SU Gerontology
S11	S4 OR S5 OR S6 OR S7 OR S8 OR S9 OR S10
S10	TI (health* OR mortalit* OR “mental health” OR “physical health” OR psychosocial*) OR SU (health* OR mortalit* OR “mental health” OR “physical health” OR psychosocial*) OR AB (health* OR mortalit* OR “mental health” OR “physical health” OR psychosocial*)
S9	SU Physical Health Assessment
S8	SU Physical Health
S7	SU Mental Health
S6	SU Mortality Rate
S5	SU Death and Dying
S4	SU Health
S3	S1 OR S2
S2	TI Voluntee* OR AB Voluntee* OR SU Voluntee*
S1	SU Volunteers

Terms used for the grey literature search will be based on the general search strategy. Combinations of terms such as “volunteer*” with terms for the population (i.e. old or elderly people) or the outcome terms (i.e. health outcomes) will be utilised.

### Description of methods used in primary research

We do not expect to find any randomised controlled trials.

Studies of the effect of voluntary work are required to have a control group for inclusion in the review. An example of a study that may be included is Musick and Wilson (2003) who compared a group of volunteers with a group of non‐volunteers of same age. They controlled for a range of socio demographic variables and in addition included two health variables as control factors. A physical health measure assessed functional impairment and the second health measure was a sum of life threatening conditions such as heart attack, stroke, lung disease, diabetes, and cancer.

### Criteria for determination of independent findings

We will take into account the unit of analysis of the studies to determine whether individuals were randomised in groups (i.e. cluster‐randomised trials), whether individuals may have undergone multiple interventions, whether there were multiple treatment groups and whether several studies are based on the same data source.

#### Cluster randomised trials

Cluster randomised trials included in this review will be checked for consistency in the unit of allocation and the unit of analysis, as statistical analysis errors can occur when they are different. When appropriate analytic methods have been used, we will meta‐analyse effect estimates and their standard errors (Higgins & Green, 2011). In cases where study investors have not applied appropriate analysis methods that control for clustering effects, we will estimate the intra‐cluster correlation (Donner, Piaggio, & Villar, 2001; Hedges, 2007b) and correct standard errors.

#### Multiple interventions groups and multiple interventions per individuals

Studies with multiple intervention groups with different individuals will be included in this review, although only intervention and control groups that meet the eligibility criteria will be used in the data synthesis. To avoid problems with dependence between effect sizes we will apply robust standard errors (Hedges, Tipton, & Johnson, 2010) and use the small sample adjustment to the estimator itself (Tipton, 2015). We will use the results in Tanner‐Smith & Tipton (2014) and Tipton (2015) to evaluate if there are enough studies for this method to consistently estimate the standard errors. See Section Data Synthesis below for more details about the data synthesis.

If there are not enough studies, we will use a synthetic effect size (the average) in order to avoid dependence between effect sizes. This method provides an unbiased estimate of the mean effect size parameter but overestimates the standard error. Random effects models applied when synthetic effect sizes are involved actually perform better in terms of standard errors than do fixed effects models (Hedges, 2007a). However, tests of heterogeneity when synthetic effect sizes are included are rejected less often than nominal.

If pooling is not appropriate (e.g., the multiple interventions and/or control groups include the same individuals), only one intervention group will be coded and compared to the control group to avoid overlapping samples. The choice of which estimate to include will be based on our risk of bias assessment. We will choose the estimate that we judge to have the least risk of bias (primarily, selection bias and in case of equal scoring the incomplete data item will be used).

#### Multiple studies using the same sample of data

In some cases, several studies may have used the same sample of data or some studies may have used only a subset of a sample used in another study. We will review all such studies, but in the meta‐analysis we will only include one estimate of the effect from each sample of data. This will be done to avoid dependencies between the “observations” (i.e. the estimates of the effect) in the meta‐analysis. The choice of which estimate to include will be based on our risk of bias assessment of the studies. We will choose the estimate from the study that we judge to have the least risk of bias (primarily, selection bias). If two (or more) studies are judges to have the same risk of bias and one of the studies (or more) uses a subset of a sample used in another study (or studies) we will include the study using the full set of participants.

#### Multiple time points

When the results are measured at multiple time points, each outcome at each time point will be analysed in a separate meta‐analysis with other comparable studies taking measurements at a similar time point. As a general guideline, these will be grouped together as follows: 1) While actively engaged in voluntary work 2) At cessation of volunteering to one year after cessation of volunteering 3) More than one year after cessation of volunteering. However, should the studies provide viable reasons for an adjusted choice of relevant and meaningful duration intervals for the analysis of outcomes, we will adjust the grouping.

### Details of study coding categories

***Selection of studies and data extraction***

Under the supervision of review authors, two review team assistants will first independently screen titles and abstracts to exclude studies that are clearly irrelevant. Studies considered eligible by at least one assistant or studies were there is insufficient information in the title and abstract to judge eligibility, will be retrieved in full text. The full texts will then be screened independently by two review team assistants under the supervision of the review authors. Any disagreement of eligibility will be resolved by the review authors. Exclusion reasons for studies that otherwise might be expected to be eligible will be documented and presented in an appendix.

The study inclusion criteria will be piloted by the review authors (see Appendix 1.1). The overall search and screening process will be illustrated in a flow diagram. None of the review authors will be blind to the authors, institutions, or the journals responsible for the publication of the articles.

Two review authors will independently code and extract data from included studies. A coding sheet will be piloted on several studies and revised as necessary (see Appendix 1.2 and 1.3).

Disagreements will be resolved by consulting a third review author with extensive content and methods expertise. Disagreements resolved by a third reviewer will be reported. Data and information will be extracted on: available characteristics of participants, intervention characteristics and control conditions, research design, sample size, risk of bias and potential confounding factors, outcomes, and results. Extracted data will be stored electronically. Analysis will be conducted using RevMan5 and Stata software.

### Assessment of risk of bias in included studies

We will assess the risk of bias using a model developed by Prof. Barnaby Reeves in association with the Cochrane Non‐Randomised Studies Methods Group (Reeves, Deeks, Higgins, & Wells, 2011).^5^ This model is an extension of the Cochrane Collaboration's risk of bias tool and covers risk of bias in non‐randomised studies that have a well‐defined control group.

The extended model is organised and follows the same steps as the risk of bias model according to the 2008‐version of the Cochrane Hand book, chapter 8 (Higgins & Green, 2008). The extension to the model is explained in the three following points:

1) The extended model specifically incorporates a formalised and structured approach for the assessment of selection bias in non‐randomised studies by adding an explicit item that focuses on confounding^6^. This is based on a list of confounders considered important and defined in the protocol for the review. The assessment of confounding is made using a worksheet, which is marked for each confounder according to whether it was considered by the researchers, the precision with which it was measured, the imbalance between groups, and the care with which adjustment was carried out (see Appendix 1.3). This assessment informs the final risk of bias score for confounding.
2) Another feature of non‐randomised studies that make them at high risk of bias is that they need not have a protocol in advance of starting the recruitment process. The item concerning selective reporting therefore also requires assessment of the extent to which analyses (and potentially, other choices) could have been manipulated to bias the findings reported, e.g., choice of method of model fitting, potential confounders considered / included. In addition, the model includes two separate yes/no items asking reviewers whether they think the researchers had a pre‐specified protocol and analysis plan.
3) Finally, the risk of bias assessment is refined, making it possible to discriminate between studies with varying degrees of risk. This refinement is achieved by the use of a 5‐point scale for certain items (see the following section *Risk of bias judgement items* for details).

The refined assessment is pertinent when considering data synthesis as it operationalizes the identification of those studies with a very high risk of bias (especially in relation to non‐randomised studies). The refinement increases transparency in assessment judgements and provides justification for excluding a study with a very high risk of bias from the data synthesis.

#### Risk of bias judgement items

The risk of bias model used in this review is based on nine items (see Appendix 1.3).

The nine items refer to:

- **sequence generation** (Judged on a low/high risk/unclear scale)
- **allocation concealment** (Judged on a low/high risk/unclear scale)
- **confounders** (Judged on a 5 point scale/unclear)
- **blinding** (Judged on a 5 point scale/unclear)
- **incomplete outcome data** (Judged on a 5 point scale/unclear)
- **selective outcome reporting** (Judged on a 5 point scale/unclear)
- **other potential threats to validity** (Judged on a 5 point scale/unclear)
- **a priori protocol** (Judged on a yes/no/unclear scale)
- **a priori analysis plan** (Judged on a yes/no/unclear scale)

In the 5‐point scale, 1 corresponds to Low risk of bias and 5 corresponds to High risk of bias. A score of 5 on any of the items assessed on the 5‐point scale translates to a risk of bias so high that the findings will not be considered in the data synthesis (because they are more likely to mislead than inform).

#### Confounding

An important part of the risk of bias assessment of non‐randomised studies is consideration of how the studies deal with confounding factors (see Appendix 1.3). Selection bias is understood as systematic baseline differences between groups which can therefore compromise comparability between groups. Baseline differences can be observable (e.g. age and gender) and unobservable (to the researcher; e.g. motivation and ‘ability’). There is no single non‐randomised study design that always solves the selection problem. Different designs represent different approaches to dealing with selection problems under different assumptions, and consequently require different types of data. There can be particularly great variations in how different designs deal with selection on unobservables. The “adequate” method depends on the model generating participation, i.e. assumptions about the nature of the process by which participants are selected into a programme. A major difficulty in estimating causal effects of voluntary work is the potential endogeneity of the individual's health condition that leads to the decision to the volunteer and if not accounted for it will yield biased estimates.

As there is no universal correct way to construct counterfactuals for non‐randomised designs, we will look for evidence that identification is achieved, and that the authors of the primary studies justify their choice of method in a convincing manner by discussing the assumption(s) leading to identification (the assumption(s) that make it possible to identify the counterfactual). Preferably the authors should make an effort to justify their choice of method and convince the reader that the only difference between an individual who volunteers and an individual who do not volunteer is not endogenous to the individuals' health conditions. The judgement is reflected in the assessment of the confounder unobservables in the list of confounders considered important at the outset (see Appendix 1.3).

In addition to unobservables, we have identified the following observable confounding factors to be most relevant: age, gender, socioeconomic status, physical health and mental health. In each study, we will assess whether these factors have been considered, and in addition we will assess other factors likely to be a source of confounding within the individual included studies.

#### Importance of pre‐specified confounding factors

The motivation for focusing on age, gender, socioeconomic status, physical health and mental health is given below.

As age in itself is related to increased health problems it is important that the comparison group is of same age as the volunteer group.

Socioeconomic status (for example education or income) is one of the strongest determinants of selection into voluntary work (Herzog & Morgan,1993) and in addition numerous studies of mortality have shown that mortality and health are also strongly related with socioeconomic status (Cutler & Lleras‐Muney, 2006; Lantz et al., 1998; Sorlie, Backlund & Keller, 1995). Women have lower mortality than men (Sorlie et al., 1995) and the socioeconomic differentials are larger for women than for men (Lantz et al., 1998). Thus, gender in itself is an important confounder.

Health is the outcome of the review and as health status (physical as well as mental) often is found to be an important predictor of volunteering among those aged 65 years or more (Wilson, 2000), it is vital that the studies demonstrate pre‐treatment group equivalence on physical and mental health.

#### Assessment

At least two review authors will independently assess the risk of bias for each included study. Any disagreements will be resolved by a third reviewer with content and statistical expertise and will be reported. We will report the risk of bias assessment in risk of bias tables for each included study in the completed review.

### Measures of treatment effect

#### Continuous outcomes

For continuous outcomes, effects sizes with 95 % confidence intervals will be calculated, where means and standard deviations are available. If means and standard deviations are not available, we will calculate SMDs from F‐ratios, t‐values, chi‐squared values and correlation coefficients, where available, using the methods suggested by Lipsey & Wilson (2001). If not enough information is yielded, the review authors will request this information from the principal investigators. Hedges' *g* will be used for estimating standardized mean differences (SMD). Any scales related to mental health (e.g. depression) and physical health (e.g. functional disability), are examples of relevant continuous outcomes in this review.

#### Dichotomous outcomes

For dichotomous outcomes, we will calculate odds ratios with 95 % confidence intervals. Physical health outcomes (e.g. mortality, serious disease (as for example a heart attack, stroke, cancer, arthritis),) are examples of relevant dichotomous outcomes in this review.

Relevant physical health outcomes may also be time until death or the onset of a serious disease in which case the effect may be measured as a hazard ratio. The hazard ratio measures the proportional change in hazard rates between individuals who volunteer and individuals who do not volunteer. The hazard rate is defined as the event rate (in the present context, the event is death or disease) at time *t* conditional on survival (no disease) until time *t* or later. The acceptable outcome measurement frequency for calculating hazard ratios in this review will be three months or less. If a study reports outcomes measured on time intervals of more than three months it will be considered to be an odds ratio.

The mental health outcomes may be reported as mean symptom scores or numbers diagnosed, i.e. numbers meeting cut off scores.

There are statistical approaches available to re‐express dichotomous and continuous data to be pooled together (Sánchez‐Meca, Marín‐Martínes & Chacón‐Moscoso, 2003). In order to calculate common metric odds ratios will be converted to SMD effect sizes using the Cox transformation. We will only transform dichotomous effect sizes to SMD if appropriate, e.g., as may be the case with the outcome depression, that can be measured with binary and continuous data.

When effect sizes cannot be pooled, study‐level effects will be reported in as much detail as possible. Software for storing data and statistical analyses will be RevMan 5.0, Excel and Stata 10.0.

### Statistical procedures and conventions

The proposed project will follow standard procedures for conducting systematic reviews using meta‐analysis techniques.

The overall data synthesis will be conducted where effect sizes are available or can be calculated, and where studies are similar in terms of the outcome measured. Meta‐analysis of both physical health outcomes and mental health outcomes will be conducted on each metric (as outlined in section ‘*Types of outcomes measures’*) separately.

As different computational methods may produce effect sizes that are not comparable, we will be transparent about all methods used in the primary studies (research design and statistical analysis strategies) and use caution when synthesizing effect sizes. Special caution will be taken concerning studies using instrumental variables (IV) and regression discontinuity (RD) to estimate a local average treatment effect (LATE) (Angrist & Pischke, 2009). These will be included, but may be subject to a separate analysis depending on the comparability between the LATE's and the effects from other studies. We will in any case check the sensitivity of our results to the inclusion of IV and RD studies. In addition we will discuss the limitation in generalisation of results obtained from these types of studies.

When the effect sizes used in the data synthesis are odds ratios, they will be log transformed before being analysed. The reason is that ratio summary statistics all have the common feature that the lowest value that they can take is 0, that the value 1 corresponds with no intervention effect, and the highest value that an odds ratio can ever take is infinity. This number scale is not symmetric. The log transformation makes the scale symmetric: the log of 0 is minus infinity, the log of 1 is zero, and the log of infinity is infinity.

Studies that have been coded with a very high risk of bias (scored 5 on the risk of bias scale) will not be included in the data synthesis.

As the intervention deal with diverse populations of participants (from different countries, facing different retirement conditions etc.), and we therefore expect heterogeneity among primary study outcomes, all analyses of the overall effect will be inverse variance weighted using random effects statistical models that incorporate both the sampling variance and between study variance components into the study level weights. Random effects weighted mean effect sizes will be calculated using 95% confidence intervals and we will provide a graphical display (forest plot) of effect sizes. Graphical displays for meta‐analysis performed on ratio scales sometimes use a log scale, as the confidence intervals then appear symmetric. This is however not the case for the software Revman 5 which we plan to use in this review^7^. The graphical displays using odds ratios and the mean effect size will be reported as a odds ratio. Heterogeneity among primary outcome studies will be assessed with Chi‐squared (Q) test, and the I‐squared, and τ‐squared statistics (Higgins, Thompson, Deeks, & Altman, 2003). Any interpretation of the Chi‐squared test will be made cautiously on account of its low statistical power.

For subsequent analyses of moderator variables that may contribute to systematic variations, we will use the mixed‐effects regression model. This model is appropriate if a predictor explaining some between‐studies variation is available but there is a need to account for the remaining uncertainty (Hedges & Pigott, 2004; Konstantopoulos, 2006).

We expect that several studies have used the same sample of data. We will review all such studies, but in the meta‐analysis we will only include one estimate of the effect from each sample of data. This will be done to avoid dependencies between the “observations” (i.e. the estimates of the effect) in the meta‐analysis. The choice of which estimate to include will be based on our quality assessment of the studies. We will choose the estimate from the study that we judge to have the least risk of bias, with particular attention paid to selection bias.

We anticipate that several studies provide results separated by for example age and/or gender. We will include results for all age and gender groups. To take into account the dependence between such multiple effect sizes from the same study, we will apply robust variance estimation (RVE) approach (Hedges et al., 2010). An important feature of this analysis is that the results are valid regardless of the weights used. For efficiency purposes, we will calculate the weights using a method proposed by Hedges et al (2010). This method assumes a simple random‐effects model in which study average effect sizes vary across studies (τ^2^) and the effect sizes within each study are equicorrelated (ρ). The method is approximately efficient, since it uses approximate inverse‐variance weights: they are approximate given that ρ is, in fact, unknown and the correlation structure may be more complex. We will calculate weights using estimates of τ^2^, setting ρ =0.80 and conduct sensitivity tests using a variety of ρ values; to asses if the general results and estimates of the heterogeneity is robust to the choice of ρ. We will use the small sample adjustment to the residuals used in RVE as proposed by Bell and McCaffrey (2002) and extended by McCaffrey, Bell and Botts et al. (2001) and by Tipton (2015). We will use the Satterthwaite degrees of freedom (Satterthwaite, 1946) for tests as proposed by Bell and McCaffrey (2002) and extended by Tipton (2015). We will use the guidelines provided in Tanner‐Smith & Tipton (2014) to evaluate if there are enough studies for this method to consistently estimate the standard errors.

If there is not a sufficient number of studies to use RVE we will conduct a data synthesis where we use a synthetic effect size (the average) in order to avoid dependence between effect sizes.

#### Moderator analysis and investigation of heterogeneity

We will investigate the following factors with the aim of explaining potential observed heterogeneity: study‐level summaries of participant characteristics (e.g. studies considering a specific gender or socioeconomic level or studies where separate effects for men/women or low/high socioeconomic status are available) and type of voluntary work (religious, educational, political etc.).

If the number of included studies is sufficient and given there is variation in the covariates, we will perform moderator analyses (multiple meta‐regression using the mixed model) to explore how observed variables are related to heterogeneity.

If there are a sufficient number of studies, we will apply the RVE approach and use approximately inverse variance weights calculated using a method proposed by Hedges et al. (2010). This technique calculates standard errors using an empirical estimate of the variance: it does not require any assumptions regarding the distribution of the effect size estimates. The assumptions that are required to meet the regularity conditions are minimal and generally met in practice. This more robust technique is beneficial because it takes into account the possible correlation between effect sizes separated by the covariates within the same study and allows all of the effect size estimates to be included in meta‐regression. We will calculate weights using estimates of τ^2^, setting ρ =0.80 and conduct sensitivity tests using a variety of ρ values; to asses if the general results is robust to the choice of ρ. We will use the small sample adjustment to the residuals used in RVE and the Satterthwaite degrees of freedom (Satterthwaite, 1946) for tests (Tipton, 2015). The results in Tipton (2015) suggests that the degrees of freedom depend on not only the number of studies but also on the type of covariates included in the meta‐regression. The degrees of freedom can be small, even when the number of studies is large if a covariate is highly unbalanced or a covariate with very high leverage is included, The degrees of freedom will vary from coefficient to coefficient. The corrections to the degrees of freedom enable us to assess when the RVE method performs well. As suggested by Tanner‐Smith & Tipton (2014) and Tipton (2015) if the degrees of freedom are smaller than four, the RVE results should not be trusted.

We will report 95% confidence intervals for regression parameters. We will estimate the correlations between the covariates and consider the possibility of confounding. Conclusions from meta‐regression analysis will be cautiously drawn and will not solely be based on significance tests. The magnitude of the coefficients and width of the confidence intervals will be taken into account as well. Otherwise, single factor subgroup analysis will be performed. The assessment of any difference between subgroups will be based on 95% confidence intervals. Interpretation of relationships will be cautious, as they are based on subdivision of studies and indirect comparisons.

In general, the strength of inference regarding differences in treatment effects among subgroups is controversial. However, making inferences about different effect sizes among subgroups on the basis of between‐study differences entails a higher risk compared to inferences made on the basis of within study differences; see Oxman & Guyatt (1992). We will therefore use within study differences where possible.

We will also consider the degree of consistence of differences, as making inferences about different effect sizes among subgroups entails a higher risk when the difference is not consistent within the studies; see Oxman & Guyatt (1992).

#### Sensitivity analysis

Sensitivity analysis will be carried out by restricting the meta‐analysis to a subset of all studies included in the original meta‐analysis and will be used to evaluate whether the pooled effect sizes are robust across components of risk of bias. We will consider sensitivity analysis for each major component of the risk of bias checklists and restrict the analysis to studies with a low risk of bias.

Sensitivity analyses with regard to research design and statistical analysis strategies in the primary studies will be an important element of the analysis to ensure that different methods produce consistent results.

#### Assessment of reporting bias

Reporting bias refers to both publication bias and selective reporting of outcome data and results. Here, we state how we will assess publication bias.

We will use funnel plots for information about possible publication bias if we find sufficient studies (Higgins & Green, 2011). However, asymmetric funnel plots are not necessarily caused by publication bias (and publication bias does not necessarily cause asymmetry in a funnel plot). If asymmetry is present, we will consider possible reasons for this.

### Treatment of qualitative research

We do not plan to include qualitative research.

### Adverse effects

It will be reported if any potential adverse effects have been evaluated in any included studies.

## Review authors

**Lead review author:** The lead author is the person who develops and co‐ordinates the review team, discusses and assigns roles for individual members of the review team, liaises with the editorial base and takes responsibility for the on‐going updates of the review.

**Table:** 

Name:	Trine Filges
Title:	Senior Researcher
Affiliation:	VIVE‐Campbell
Address:	Herluf Trollesgade 11
City, State, Province or County:	Copenhagen
Postal Code:	1052
Country:	Denmark
Phone:	45 33480926
Email:	tif@vive.dk
**Co‐authors:**	
Name:	Anu Siren
Title:	Senior researcher
Affiliation:	VIVE‐Campbell
Address:	Herluf Trollesgade 11
City, State, Province or County:	Copenhagen
Postal Code:	1052
Country:	Denmark
Phone:	+45 33697753
Email:	anu@vive.dk
	
Name:	Torben Fridberg
Title:	Senior researcher
Affiliation:	VIVE‐Campbell
Address:	Herluf Trollesgade 11
City, State, Province or County:	Copenhagen
Postal Code:	1052
Country:	Denmark
Phone:	+45 33480847
Email:	tf@vive.dk
	
Name:	Bjørn Christian Viinholt Nielsen
Title:	Librarian/Information Specialist
Affiliation:	VIVE‐Campbell
Address:	Herluf Trollesgade 11
City, State, Province or County:	Copenhagen
Postal Code:	1052
Country:	Denmark
Phone:	+45 22442827
Email:	bcn@vive.dk

## Roles and responsibilities

- Content: Anu Siren and Torben Fridberg
- Systematic review methods: Trine Filges
- Statistical analysis: Trine Filges and Torben Fridberg
- Information retrieval: Bjørn Christian Viinholt Nielsen

## Sources of support

VIVE‐Campbell.

## Declarations of interest

None

## Preliminary timeframe

Approximate date for submission of the systematic review is 1 year after protocol approval

## Plans for updating the review

Once completed, we plan to update the review with a frequency of two years. Trine Filges will be responsible.

## AUTHOR DECLARATION

### Authors' responsibilities

By completing this form, you accept responsibility for preparing, maintaining and updating the review in accordance with Campbell Collaboration policy. The Campbell Collaboration will provide as much support as possible to assist with the preparation of the review.

A draft review must be submitted to the relevant Coordinating Group within two years of protocol publication. If drafts are not submitted before the agreed deadlines, or if we are unable to contact you for an extended period, the relevant Coordinating Group has the right to de‐register the title or transfer the title to alternative authors. The Coordinating Group also has the right to de‐register or transfer the title if it does not meet the standards of the Coordinating Group and/or the Campbell Collaboration.

You accept responsibility for maintaining the review in light of new evidence, comments and criticisms, and other developments, and updating the review at least once every five years, or, if requested, transferring responsibility for maintaining the review to others as agreed with the Coordinating Group.

### Publication in the Campbell Library

The support of the Coordinating Group in preparing your review is conditional upon your agreement to publish the protocol, finished review, and subsequent updates in the Campbell Library. The Campbell Collaboration places no restrictions on publication of the findings of a Campbell systematic review in a more abbreviated form as a journal article either before or after the publication of the monograph version in Campbell Systematic Reviews. Some journals, however, have restrictions that preclude publication of findings that have been, or will be, reported elsewhere and authors considering publication in such a journal should be aware of possible conflict with publication of the monograph version in Campbell Systematic Reviews. Publication in a journal after publication or in press status in Campbell Systematic Reviews should acknowledge the Campbell version and include a citation to it. Note that systematic reviews published in Campbell Systematic Reviews and co‐registered with the Cochrane Collaboration may have additional requirements or restrictions for co‐publication. Review authors accept responsibility for meeting any co‐publication requirements.

**I understand the commitment required to undertake a Campbell review, and agree to publish in the Campbell Library. Signed on behalf of the authors**:

**Table:** 

**Form completed by: T. Filges**	**Date: 15 October 2018**

## Data extraction

**Table:** 

**Authors**
**Journal**
**Year**
**Country**
**Time period covered by data**
**Type of voluntary work**
**Participation characteristics (age, gender, education, ethnicity)**
**Hours of voluntary work**
**Type of data used (register, questionnaire, other (specify))**
**Sampling frequency**
**Time interval the outcome measure is based on (if different from sampling frequency)**
**Sample size (Treatment/control)**

### Outcome measures

Instructions: Please enter outcome measures in the order in which they are described in the report. Note that a single outcome measure can be completed by multiple sources and at multiple points in time (data from specific sources and time‐points will be entered later).

**Table:** 

#	Outcome & measure	Reliability & Validity	Format	Direction	Source	Pg# & notes
1		Info from: Other samples This sample Unclear Info provided:	Dichotomy Continuous Time‐to‐event	High score or event is Positive Negative Can't tell	Questionnaire Admin data Other (specify) Unclear	

* Repeat as needed

### DICHOTOMOUS OUTCOME DATA

**Table:** 

OUTCOME	TIME POINT (s) (record exact time, there may be more than one, record them all)	SOURCE	VALID Ns	CASES	NON‐CASES	STATISTICS	Pg. # & NOTES
		Questionnaire Admin data Other (specify) Unclear	Intervention	Intervention	Intervention	RR (risk ratio) OR (odds ratio) SE (standard error) 95% CI DF exact p value Chi2 Other	
		
			Comparison	Comparison	Comparison		
		

Repeat as needed

### TIME‐TO‐EVENT OUTCOME DATA

**Table:** 

OUTCOME	TIME POINT (s) (record exact time, there may be more than one, record them all)	SOURCE	Method of estimation			STATISTICS	Pg. # & NOTES
		Questionnaire Admin data Other (specify) Unclear	Non‐parametric Semi‐parametric Parametric			HR (hazard ratio) SE (standard error) 95% CI DF exact p value Chi2 Other	
	
	
	

Repeat as needed

### CONTINUOUS OUTCOME DATA

**Table:** 

OUTCOME	TIME POINT (s) (record exact time, there may be more than one, record them all)	SOURCE (specify)	VALID Ns	Means	SDs	STATISTICS	Pg. # & NOTES
		Questionnaire Admin data Other (specify) Unclear	Intervention	Intervention	Intervention	P, t or F Df ES Covariates Other	
		
			Comparison	Comparison	Comparison		
		

*Repeat as needed

## Assessment of risk of bias

**Risk of bias table**

**Table:** 

**Item**	**Judgement** ^a^	**Description** (quote from paper, or describe key information)
1. Sequence generation		
2. Allocation concealment		
3. Confounding^b^, ^c^		
4. Blinding?^b^		
5. Incomplete outcome data addressed?^b^		
6. Free of selective reporting?^b^		
7. Free of other bias?		
*8. A priori* protocol?^d^		
*9. A priori* analysis plan?^e^		

a Some items on low/high risk/unclear scale (double‐line border), some on 5 point scale/unclear (single line border), some on yes/no/unclear scale (dashed border). For all items, record “unclear” if inadequate reporting prevents a judgement being made.

b For each outcome in the study.

c This item is only used for NRCTs and NRSs. It is based on list of confounders considered important at the outset and defined in the protocol for the review (*assessment against worksheet*).

d Did the researchers write a protocol defining the study population, intervention and comparator, primary and other outcomes, data collection methods, etc. in advance of starting the study?

e Did the researchers have an analysis plan defining the primary and other outcomes, statistical methods, subgroup analyses, etc. in advance of starting the study?

### Risk of bias tool

#### Studies for which RoB tool is intended

The risk of bias model was developed by Prof. Barnaby Reeves in association with the Cochrane Non‐Randomised Studies Methods Group.^8^ This model, an extension of the Cochrane Collaboration's risk of bias tool, covers risk of bias in both randomised controlled trials (RCTs and QRCTs) and in non‐randomised studies (NRCTs and NRSs).

The point of departure for the risk of bias model is the Cochrane Handbook for Systematic Reviews of interventions (Higgins & Green, 2008). The existing Cochrane risk of bias tool needs elaboration when assessing non‐randomised studies because, for non‐randomised studies, particular attention should be paid to selection bias / risk of confounding. Additional item on confounding is used only for non‐randomised studies (NRCTs and NRSs) and is not used for randomised controlled trials (RCTs and QRCTs).

### Assessment of risk of bias

Issues when using modified RoB tool to assess included non‐randomised studies:

- Use existing principle: score judgment and provide information (preferably direct quote) to support judgment
- Additional item on confounding used only for non‐randomised studies (NRCTs and NRSs).
- 5‐point scale for some items (distinguish “unclear” from intermediate risk of bias).
- Keep in mind the general philosophy – assessment is not about whether researchers could have done better but about risk of bias; the assessment tool must be used in a standard way whatever the difficulty / circumstances of investigating the research question of interest and whatever the study design used.
- Anchors: “1/No/low risk” of bias should correspond to a high quality RCT. “5/high risk” of bias should correspond to a risk of bias that means the findings should not be considered (too risky, too much bias, more likely to mislead than inform)

1. Sequence generation
  - Low/high/unclear RoB item
  - Always high RoB (not random) for a non‐randomised study
  - Might argue that this item redundant for NRS since always high – but important to include in RoB table (‘level playing field’ argument)
2. Allocation concealment
  - Low/high/unclear RoB item
  - Potentially low RoB for a non‐randomised study, e.g. quasi‐randomised (so high RoB to sequence generation) but concealed (reviewer judges that the people making decisions about including participants didn't know how allocation was being done, e.g. odd/even date of birth/hospital number)
3. RoB from confounding (additional item for NRCT and NRS; assess for each outcome)
  - Assumes a pre‐specified list of potential confounders defined in the protocol
  - Low(1) / 2 / 3 / 4 / high(5) / unclear RoB item
  - Judgment needs to factor in:
    ○ proportion of confounders (from pre‐specified list) that were considered
    ○ whether most important confounders (from pre‐specified list) were considered
    ○ resolution/precision with which confounders were measured
    ○ extent of imbalance between groups at baseline
    ○ care with which adjustment was done (typically a judgment about the statistical modeling carried out by authors)
  - Low RoB requires that all important confounders are balanced at baseline (not primarily/not only a statistical judgment OR measured ‘well’ and ‘carefully’ controlled for in the analysis.
  Assess against pre‐specified worksheet. Reviewers will make a RoB judgment about each factor first and then ‘eyeball’ these for the judgment RoB table.
4. RoB from lack of blinding (assess for each outcome, as per existing RoB tool)
  - Low(1) / 2 / 3 / 4 / high(5) / unclear RoB item
  - Judgment needs to factor in:
    ○ nature of outcome (subjective / objective; source of information)
    ○ who was / was not blinded and the risk that those who were not blinded could introduce performance or detection bias
    ○ see Ch.8
5. RoB from incomplete outcome data (assess for each outcome, as per existing RoB tool)
  - Low(1) / 2 / 3 / 4 / high(5) / unclear RoB item
  - Judgment needs to factor in:
    ○ reasons for missing data
    ○ whether amount of missing data balanced across groups, with similar reasons
    ○ whether censoring is less than or equal to 25% and taken into account
    ○ see Ch.8
6. RoB from selective reporting (assess for each outcome, NB different to existing Ch.8 recommendation)
  - Low(1) / 2 / 3 / 4 / high(5) /unclear RoB item
  - Judgment needs to factor in:
    ○ existing RoB guidance on selective outcome reporting (see Ch.8)
    ○ also, extent to which analyses (and potentially other choices) could have been manipulated to bias the findings reported, e.g. choice of method of model fitting, potential confounders considered / included
    ○ look for evidence that there was a protocol in advance of doing any analysis / obtaining the data (difficult unless explicitly reported); NRS very different from RCTs. RCTs must have a protocol in advance of starting to recruit (for REC/IRB/other regulatory approval); NRS need not (especially older studies)
    ○ Hence, separate yes/no items asking reviewers whether they think the researchers had a pre‐specified protocol and analysis plan.
7. RoB from other bias (assess for each outcome, NB different to existing Ch.8 recommendation)
  - Low(1) / 2 / 3 / 4 / high(5) /unclear RoB item
  - Judgment needs to factor in:
    ○ existing RoB guidance on other potential threats to validity (see Ch.8)
    ○ also, assess whether suitable cluster analysis is used (e.g. cluster summary statistics, robust standard errors, the use of the design effect to adjust standard errors, multilevel models and mixture models), if assignment of units to treatment is clustered

**Confounding workshee** **t**

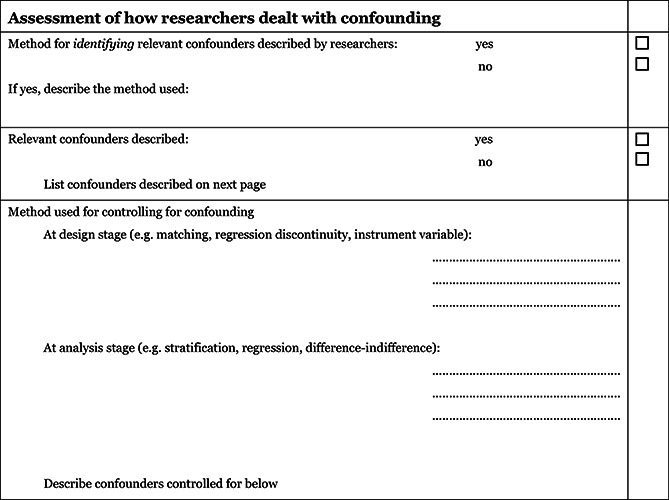

**Confounders described by researchers**

Tick (yes[0]/no[1] judgment) if confounder considered by the researchers [Cons'd?]

Score (1[good precision] to 5[poor precision]) precision with which confounder measured

Score (1[balanced] to 5[major imbalance]) imbalance between groups

Score (1[very careful] to 5[not at all careful]) care with which adjustment for confounder was carried out

**Table:** 

**Confounder**	Considered	Precision	Imbalance	Adjustment
Gender	□	□	□	□
Age	□	□	□	□
SES	□	□	□	□
Pre physical health	□	□	□	□
Pre mental health	□	□	□	□
Unobservables^9^	□	Irrelevant	□	□
Other:	□	□	□	□

### User guide for unobservables

Selection bias is understood as systematic baseline differences between groups and can therefore compromise comparability between groups. Baseline differences can be observable (e.g. age and gender) and unobservable (to the researcher; e.g. motivation and ‘ability’). There is no single non‐randomised study design that always solves the selection problem. Different designs solve the selection problem under different assumptions and require different types of data. Especially how different designs deal with selection on unobservables varies. The “right” method depends on the model generating participation, i.e. assumptions about the nature of the process by which participants are selected into a programme.

As there is no universal correct way to construct counterfactuals we will assess the extent to which the identifying assumptions (the assumption that makes it possible to identify the counterfactual) are explained and discussed (preferably the authors should make an effort to justify their choice of method). We will look for evidence that authors using e.g. (this is NOT an exhaustive list):

**Natural experiments:**

Discuss whether they face a truly random allocation of participants and that there is no change of behaviour in anticipation of e.g. policy rules.

**Instrument variable (IV):**

Explain and discuss the assumption that the instrument variable does not affect outcomes other than through their effect on participation.

**Matching (including propensity scores):**

Explain and discuss the assumption that there is no selection on unobservables, only selection on observables.

**(Multivariate, multiple) Regression:**

Explain and discuss the assumption that there is no selection on unobservables, only selection on observables. Further discuss the extent to which they compare comparable people.

**Regression Discontinuity (RD):**

Explain and discuss the assumption that there is a (strict!) RD treatment rule. It must not be changeable by the agent in an effort to obtain or avoid treatment. Continuity in the expected impact at the discontinuity is required.

**Difference‐in‐difference (Treatment‐control‐before‐after):**

Explain and discuss the assumption that outcomes of participants and nonparticipants evolve over time in the same way.

## Footnotes

1 Australia, Austria, Belgium, Canada, Chile, Czech Republic, Denmark, Estonia, Finland, France, Germany, Greece, Hungary, Iceland, Ireland, Israel, Italy, Japan, Korea, Luxembourg, Mexico, Netherlands, New Zealand, Norway, Poland, Portugal, Slovak Republic, Slovenia, Spain, Sweden, Switzerland, Turkey, United Kingdom and United States.

2 For the purpose of this review, ‘Europe’ is defined as the 28 European Union (EU) countries, namely: Belgium, France, Italy, Luxembourg, Netherlands, Germany, Denmark, Ireland, United Kingdom, Greece, Spain, Portugal, Austria, Sweden, Finland, Malta, Cyprus, Slovenia, Estonia, Latvia, Lithuania, Poland, the Czech Republic, Slovakia, Hungary, Romania, Bulgaria.

3 See note 1

4 Currently there are more than 100 local groups in Denmark

5 This risk of bias model was introduced by Prof. Reeves at a workshop on risk of bias in non‐randomised studies at SFI Campbell, February 2011. The model is a further development of work carried out in the Cochrane Non‐Randomised Studies Method Group (NRSMG).

6 See next page for an explanation of the terms selection bias and confounding.

7 If we apply robust variance estimation the analysis will be conducted in STATA as robust variance estimation is not implemented in Revman 5.

8 This risk of bias model was introduced by Prof. Reeves at a workshop on risk of bias in non‐randomised studies at SFI Campbell, February 2011. The model is a further development of work carried out in the Cochrane Non‐Randomised Studies Method Group (NRSMG).

9 See user guide for unobservables

## First and second level screening

First level screening is on the basis of titles and abstracts. Second level is on the basis of full text

Reference id. No. :

Study id. No.:

Reviewers initials:

Source:

Year of publication:

Duration of study:

Country/countries of origin

Author

The study will be excluded if one or more of the answers to question 1‐3 are ‘No’. If the answers to question 1 to 3 are ‘Yes' or ‘Uncertain’, then the full text of the study will be retrieved for second level eligibility. All unanswered questions need to be posed again on the basis of the full text. If not enough information is available, or if the study is unclear, the author of the study will be contacted if possible.

**First level screening questions are based on titles and abstracts**

1. Are the participants' individuals who do formal voluntary work?
  Yes ‐ include
  No – if no then stop here and exclude
  Uncertain ‐ include

Question 1 guidance:

This includes all types of formal voluntary work described as on‐going, planned, helping behaviour that intend to increase the well‐being of strangers, offers no monetary compensation, and typically occurs within an organizational context (religious, educational or health organisations, political groups, senior citizen groups or related organizations). Informal ways of helping friends, neighbours, or relatives, such as running errands, providing transportation etc., which are typically motivated by an obligation to help intimate others, will be excluded.

2. Does the study include volunteers aged 65 and more?
  Yes ‐ include
  No – if no then stop here and exclude
  Uncertain ‐ include

Question 2 guidance:

Studies where the majority of participants are aged 65 or over, or where results are shown for subgroups of participants aged 65 or over, will be included.

3. Is this study a primary quantitative study?
  Yes ‐ include
  No – if no then stop here and exclude
  Uncertain ‐ include

Question 3 guidance:

We are only interested in primary quantitative studies, where the authors have analyzed the data. We are not interested in theoretical papers on the topic or surveys/reviews of studies of the topic. (This question may be difficult to answer on the base of titles and abstracts alone.)

**Second level screening questions based on full text**

4. Does the study estimate an effect, using a control group or using an estimated counterfactual?
  Yes ‐ include
  No – if no then stop here and exclude
  Uncertain ‐ include

Question 4 guidance

E.g. 1) Randomised controlled trials including cluster randomisation and quasi randomised controlled study designs (i.e. participants are allocated by means such as alternate allocation, person's birth date, the date of the week or month, case number or alphabetical order), 2) non randomised controlled study designs (i.e. quasi‐experimental designs) such as controlled two group study designs or 3) study designs based on observational data, where the effect is estimated by statistical methods.

5. Does the study examine physical health (including mortality) or mental health?
  Yes – include
  No – if no then stop here and exclude
  Uncertain – include

Question 5 guidance:

Examples of physical health outcomes include mortality, time until the onset of a serious disease (as for example a heart attack, stroke, cancer, arthritis), functional disability (measured by a standardized physical ability measure such as a difficulties in activities of daily living score (ADLs, see Katz et al., 1963)), or a difficulties in instrumental activities of daily living score (IADLs, see Lawton and Brody, 1969).

Examples of mental health outcomes include depression, anxiety and mental health‐related disability measured by standardized psychological symptom measures such as the Center for Epidemiological Studies Depression Scale (CES‐D), the Hopkins Symptom Checklist and the Medical Outcomes Study – Short Form.
